# Motivating Seasonal Influenza Vaccination and Cross-Promoting COVID-19 Vaccination: An Audience Segmentation Study among University Students

**DOI:** 10.3390/vaccines9121397

**Published:** 2021-11-25

**Authors:** Daisy Lee, Sharyn Rundle-Thiele, Gabriel Li

**Affiliations:** 1 School of Professional Education and Executive Development, The Hong Kong Polytechnic University, Hong Kong; tlgab.li@speed-polyu.edu.hk; 2 Social Marketing @ Griffith, Griffith University, Nathan, QLD 4111, Australia; s.rundle-thiele@griffith.edu.au

**Keywords:** seasonal influenza vaccination, COVID-19 vaccination, segmentation, social marketing, health promotion

## Abstract

In the response to the coronavirus pandemic, much attention has been invested in promoting COVID-19 vaccination. However, the impact of seasonal influenza should not be neglected, particularly during the winter influenza surge. Currently, most influenza vaccination campaigns target at healthcare workers or high-risk population groups, while COVID-19 vaccination programmes are targeting the whole population as a single homogeneous group. There is limited research on the promotion of influenza vaccination for university students who study, live, and socialise in close contact with a large variety of people on campus, resulting in a low vaccination rate among this underserved group. Thus, a vaccination programme tailored for university students should be developed to increase protection against influenza-like illnesses and complications, and to help achieve herd immunity across populations who spread viruses. WHO has advocated the potential value of social marketing in vaccination campaigns and highlighted the need for audience segmentation as a major prerequisite component of intervention design. This study aims to identify distinct and homogeneous groups of university students based on sociodemographic, psychographic, and behavioural attributes to inform interventions. Two-step cluster analysis was applied in a sample size of 530 university students and revealed four segments that demonstrate statistically significant differences in their attitudes, behaviours, intentions, and responses to promotion messages about seasonal influenza and COVID-19 vaccination. The findings provide a detailed understanding of segment characteristics among university students that can be applied to develop an effective social marketing campaign that can motivate influenza vaccination and cross-promote uptake of the COVID-19 vaccine.

## 1. Introduction

Seasonal influenza, an acute illness of the respiratory tract and highly infectious disease caused by influenza viruses, is a severe health threat worldwide. Every year, half a million deaths and five million cases of critical illness around the globe are estimated to be a consequence of seasonal influenza [1]. Compared to the COVID-19 virus, seasonal influenza might have a smaller fraction of critical cases and mortality rate, but the influenza virus can be transmitted faster than the COVID-19 virus [2]. While significant energy has been expended on the COVID-19 pandemic, seasonal influenza requires ongoing attention as it has been a global health issue for centuries.

Vaccination is identified as the most effective measure to prevent seasonal influenza and influenza-related respiratory illnesses [3]; however, many people choose not to be vaccinated for various reasons. Even in the United States, where vaccination against influenza is relatively common, only slightly more than half of the population is covered [4]. For the global population, seasonal influenza vaccination rates among university students are lower than other population groups [5] but spreading rates are higher [6]. Promoting vaccination against seasonal influenza among this segment of the population is essential to combat the disease.

While most university students do not belong to any high-risk groups for influenza complications, once infected, they suffer certain consequences that are preventable. Seasonal influenza can cause a range of symptoms, such as fever and cough, that can last for many weeks [7]. Influenza-like illnesses also obstruct the personal development of students as they have lower class attendance and impaired academic performance and internship productivity [8]. In addition, the illnesses can also interrupt their daily routines so that they are unable to participate in social or extracurricular activities, which can have negative consequences on wellbeing.

University settings are recognised as a hotbed for the spread of influenza and other viruses. A university lecture inside a compact indoor area could be attended by hundreds of students. The density of the classroom setting coupled with the duration of a lecture favours the spread of influenza. The infected students can spread the virus among their peers by coughing, sneezing, or talking [6]. Factoring in the close social and living spaces of university students, outbreaks of influenza on university campuses are prevalent due to the highly infectious nature of the virus and the conditions facilitating the spread [9]. An outbreak at a campus has the potential to spread into the community; therefore, quarantining the campus could be critical to block transmission from the student population to the general public [10]. Similarly, preventing the outbreak of seasonal influenza among university students through vaccination can minimise community-level spread.

Promoting vaccination against seasonal influenza among university students is an indispensable step in combating the virus. Immunity studies and statistics demonstrate that a substantial reduction in influenza-like illnesses among low-risk population groups can significantly reduce transmission in the community and provide increased protection for the high-risk individuals [8,11]. Increasing vaccine uptake among university students could concurrently protect them from the virus and minimise their chances of transmitting the virus to vulnerable family members and high-risk individuals in the community. ‘Herd immunity’ could be achieved if vaccination rates exceed 30% of the population, hence protecting both vaccinated and unvaccinated individuals in the community from an influenza outbreak [11]. Therefore, promoting vaccine uptake among university students plays a role that should not be overlooked in preventing seasonal influenza.

Policy makers around the globe are struggling to combat the coronavirus pandemic by offering free COVID-19 vaccines to their citizens. With exceptionally low acceptance and vaccination rate of COVID-19 among university students, many studies sought to explore interventions that may boost COVID-19 vaccination among this group of young and healthy individuals [12,13], aiming to attain herd immunity in the community and resume on-campus activities. Promoting COVID-19 vaccines to students who have higher vaccine confidence and lower vaccine hesitancy is found to be one of the effective ways to boost the vaccination rate [12]. However, it may be difficult to identify which individuals are of higher confidence and acceptancy towards COVID-19 vaccines. Thus, COVID-19 vaccination promotion activities are currently targeting everyone using the same promotion mechanics. By understanding attitude towards influenza vaccines and influenza vaccination behaviour in recent years, university clinics may cross-promote COVID-19 vaccination to those who receive influenza vaccination and have higher vaccine acceptance.

### The Importance of Segmentation in Vaccination Promotion

The World Health Organization (WHO) stresses the necessity of segmenting and prioritising the target audiences when developing effective vaccine uptake promotion and communication programmes [14]. Segmentation acknowledges differences within populations and seeks to organise people into groups who are most alike, enabling differences in the way groups think, feel, and behave to be clearly illuminated. By understanding group differences, consideration can be given to what messages are promoted, when and where to capture the attention of more people through delivering what they need to hear.

Vaccine hesitancy arises from various reasons or emotions. It would be illogical to expect that a one-size-fits-all approach could alter behaviours among one heterogeneous population [15]. Segmentation is therefore needed to increase vaccination uptake. By breaking one heterogeneous population into different homogeneous subgroups who exhibit similar apprehension or doubts, segmentation helps lay the foundation for the development of effective communication or intervention capable of dismissing specific doubts or fears [16].

The concept of segmentation has existed in marketing literature for almost half a century. Instead of targeting a large population, marketing segmentation analysis identifies similar characteristics shared among a sub-group, hence categorising them into segments [17]. This commercial marketing concept was embraced by social marketers and has become one of the six benchmarks that qualify an approach as social marketing, along with behaviour change, formative research, exchange, marketing mix (i.e., using all four Ps including product, price, place, and promotion), and competition [18,19]. While the WHO advocates exploring the potential value of the social marketing approach to address low vaccination rates [20], segmentation of target audiences is an important benchmark criterion for developing social marketing practice to effectively influence heterogeneous audience segments [17]. Segmentation can be conducted using four bases: demographic, geographic, psychographic, and behavioural. While the first two bases are the most commonly used, their inability to anticipate behaviour makes them less important regarding the segment formation process than psychographic and behavioural bases [19].

Segmentation has already been applied by scholars attempting to promote influenza vaccine uptake. Lau et al. [16] measured vaccine-related behaviours and attitudes along with demography among Hong Kong healthcare workers to divide nurses into segments of various levels of hesitancy, proposing unique strategies for each segment that pinpoint the respective barriers to seasonal influenza vaccination. Similarly, in addition to the demographic base, Ramanadhan et al. [15] also used psychographic (e.g., attitude towards vaccine) and behavioural (e.g., news consumption behaviour) bases to categorise vaccine-hesitant individuals into three clusters and suggested corresponding, tailored communication strategies. Both Lau, Lee and Wong [16] and Ramanadhan et al. [15] demonstrate how segmentation can be used to gain a better understanding of the subgroups among the population, each with particular characteristics that lead to vaccine uptake intentions and behaviours. Lau et al. [16] study on healthcare workers further suggests that heterogeneous segments could still be identified among nurses although they are seemingly homogeneous with similar socioeconomic status, thus reinforcing the significance of employing segmentation before developing a communication strategy to promote vaccine uptake. While the WHO [14] advocates the use of segmentation to tailor immunisation programmes, no prior research has been directed towards understanding how segmentation can be applied to deliver the information needed to inform planning for a campaign aiming to motivate university students to receive seasonal influenza vaccinations.

This paper aims to employ audience segmentation analysis to identify how university students can be divided into various key segments according to sociodemographic (personal, academic, family, and health particulars), psychographic (level of involvement and attitude towards seasonal influenza vaccine), and behaviour (vaccination behaviour, information search behaviour, and information exposure) variables. A recent public health communication study conducted by Ihm and Lee [21] demonstrates that engagement with online media resources should be used to segment the generation born in the digital era. Based on the distinctive characteristics of each segment, this paper also examines possible seasonal influenza vaccine promotion and communication tactics to understand which messages may be used to motivate seasonal flu vaccination among university students. Seasonal influenza vaccination promotion messages and influencers identified from 5 online focus group studies with 20 undergraduate students are concept tested with the identified segments to inform future social marketing programme design.

This study was conducted two months after the Hong Kong government began to administer free COVID-19 vaccines, and students were informed that COVID-19 vaccination may be required for the resumption of face-to-face classes in the 2021–2022 academic year. Thus, COVID-19 vaccination behaviours, attitudes, and intentions were also explored to determine if the segmentation study of seasonal influenza vaccination provides any insights into the development of a COVID-19 vaccination promotion programme. To add value to the current efforts to combat the COVID-19 pandemic, this study also provides a brief review of possible cross-promotional tactics to influence the uptake of COVID-19 vaccines targeting different influenza vaccination audience segments.

## 2. Materials and Methods

### 2.1. Design and Participants

This study used a cross-sectional online survey conducted in the self-financing, full-time tertiary education unit of a local university in Hong Kong. Convenience sampling was used, and an email invitation to participate in the study was sent to each of the 11,000 students in May 2021 following the winter seasonal influenza peak of 2020–2021. The email explained the research objectives, nature of the study, incentive for participation, and provided the link to participate in the online survey. A HKD50 supermarket voucher was offered to each student who successfully completed the online survey to incentivise students to participate, resulting in 530 valid responses collected from all major academic disciplines, including sciences and engineering, health-related studies, business and marketing, language and translation, arts and humanities, and social sciences.

### 2.2. Measurement

Questionaires are presented in Supplementary Materials File S1. The questionnaire, constructed bilingually in Chinese and English, consisted of six sections: (i) sociodemographic and academic details; (ii) personal health conditions, history of influenza-like illness, vaccination behaviours of family members, and experiences associated with other vaccines; (iii) seasonal influenza vaccine-related behaviours, attitudes, and intentions; (iv) seasonal influenza-related information search, exposure, and trust; (v) message and influencer in motivating seasonal influenza vaccination; and (vi) COVID-19 vaccination behaviours, attitudes, and intentions.

Section (i) recorded participants’ age, sex, area of study, year of study, GPA, and whether the respondent lives with or is in frequent contact with high-risk population groups including elderly people, young children, and people with chronic illness (yes or no) [16].

Section (ii) recorded participants’ personal health conditions, including whether the respondents ‘have chronic diseases’ (yes or no), ‘had voluntarily received other vaccines (e.g., HPV or hepatitis vaccines)’ (yes or no), ‘had encountered severe allergic reaction to a vaccine injection’ (yes or no), and ‘the frequency of having flu-related illness in past 5 years’ (every year, 3–4 years, 1–2 years, never) [16].

Section (iii) measured seasonal influenza vaccine-related behaviours, attitudes, and intentions using validated constructs and items. Influenza vaccine-related behaviours measured influenza vaccination in the 2020–2021 season (yes or no) and frequency of influenza vaccination in the past five years (every year, 3–4 years, 1–2 years, never). Influenza vaccine-related attitudes measured components of the 5Cs model (confidence, complacency, convenience, calculation, and collective responsibility) that affect the psychological factors underpinning vaccination decisions [22]. Betsch et al. [22] extend the globally adopted 3Cs model of vaccine hesitancy (confidence, complacency, convenience) and the fourth barrier of influenza vaccination intention and behaviour (calculation) to include collective responsibility as the fifth reason for vaccination uptake [1]. All questions in this section were measured using 7-point Likert scale (1 = strongly disagree; 7 = strongly agree). Four items measured respondents’ confidence in the safety of influenza vaccines and the system that delivers the vaccines by rating their level of agreement for the sentences ‘flu vaccine is safe’, ‘flu vaccine is effective in protecting individuals from getting influenza’, ‘confident that government and healthcare professionals could decide the best interest of the community when recommending flu vaccines’, and ‘concerned about side-effects of seasonal flu vaccines’ (reverse scale). A higher score means more confidence in seasonal influenza vaccines and stronger positive attitudes towards influenza vaccination. 

Three items measured respondents’ complacency to examine the perceived risks of diseases that can be preventable through seasonal influenza vaccination. The items are: ‘seasonal flu vaccination is necessary for my immune system to be strong enough to fight against the influenza virus’, ‘seasonal flu vaccination is not a priority for me because influenza is not a serious illness but just a routine and mild illness’, and ‘if I get a flu, the illness will greatly affect my academic study, social life or job performance’ (reverse scale). A higher score means lower perceived risks of influenza-like illness and lower intentions to get vaccinated. 

Two items measured convenience by asking respondents to rate their level of agreement with ‘the location to receive seasonal flu vaccinations is convenient’ and ‘unavailability of free seasonal flu vaccine is a main barrier to vaccination’ (reverse scale). A higher score means that the availability and affordability of influenza vaccines is high, resulting in stronger positive attitudes and intentions to vaccinate. 

Two items measured calculation by asking respondents to rate the sentences ‘when I think about getting vaccinated, I weigh the benefits and risks to make the best decision possible’ and ‘the benefits of having seasonal flu vaccines are higher than the risks’. A higher score means that respondents do not have strong, pre-determined attitudes towards influenza vaccination and that decisions to get vaccinated are based on careful evaluation of subjective factors. 

Collective responsibility was measured using two items: ‘I get vaccinated because I can also protect people with a weaker immune system’ and ‘seasonal flu vaccination is a collective action to prevent the spread of influenza’. A higher score means greater willingness to protect others through receiving an influenza vaccination. 

In addition to the 5Cs model factors, level of involvement in the decision making of receiving a seasonal influenza vaccine was measured using the personal involvement inventory developed by Zaichkowsky [23]. Respondents were asked to rate whether receiving seasonal influenza vaccination is ‘unimportant/important’, ‘of no concern/of concern to me’, ‘means nothing to me/means a lot to me’, and ‘does not matter/matters to me’ using 7-point semantic differential scales. 

Finally, seasonal influenza vaccination intention was measured by the intention to be vaccinated in the coming influenza season (2021–2022) using a 7-point Likert scale (1 = definitely would not; 7 = definitely would).

Section (iv) measured seasonal influenza-related information searches, exposure, and trust. Participants’ information-seeking behaviour and level of attention paid to influenza vaccine-related information exposed to them were measured using a 7-point Likert scale (1 = strongly disagree; 7 = strongly agree). Online and offline channel sources from which most seasonal influenza-related information was actively searched for or received were recorded (yes/no). Trust in sources from which most influenza information was received and usual seasonal influenza information search channels were measured using a 7-point Likert scale (1 = do not trust at all; 7 = completely trust).

Section (v) measured respondents’ feeling towards promotional messages and influencers in motivating them to get a seasonal influenza vaccination. In qualitative focus group studies with 20 students prior to this survey, a list of promotional messages and potential influencers that may be used in promoting influenza vaccine uptake among university students was identified for concept testing in this study.

Section (vi) explored COVID-19 vaccination behaviours, attitudes, and intentions. The questions and measurement scales used in Section 3 were repeated in this section, but the vaccine was revised from seasonal influenza vaccine to COVID-19 vaccine. This cross-sectional survey was conducted in May 2021 when the Hong Kong government had administered free COVID-19 vaccination in Hong Kong for approximately 10 weeks. At the time, the universities in Hong Kong were considering resuming face-to-face teaching in September 2021 under the condition that all teachers and students had received COVID-19 vaccinations.

## 3. Results

This study employed two-step cluster analysis to identify homogeneous segments. This was considered the most appropriate method for several reasons [24]. First, due to the exploratory nature of this segmentation study, the number of clusters was not pre-determined but estimated using an SPSS algorithm based on indexes of fit. Second, unlike other clustering methods, two-step cluster analysis supports the formation of segments based on a combination of categorical and continuous variables which offers the flexibility to simultaneously process a diverse range of variables in segment formation [25]. Third, two-step cluster analysis has been widely used in prior segmentation studies in social marketing [19], influenza vaccine hesitancy [16], and health behaviours [26,27].

SPSS used auto-clustering to determine the optimal number of clusters by grouping the cases into pre-clusters based on log-likelihood distance and followed by a standard hierarchical clustering algorithm to reduce the pre-clustered groups to the optimal number of clusters using Schwarz’s Bayesian criterion (BIC). The auto-clustering statistics are presented in Table 1. The SPSS algorithm provided a four-cluster solution at which both the BIC and the change in BIC between clusters becomes smallest. Two-step cluster analysis produced four clusters with the silhouette measure of cohesion and separation of 0.6, indicating good cluster quality [25]. Table 2 presents the size of each segment. 

Four segmentation variables (seasonal influenza vaccination behaviour in the current season; personal involvement; information search behaviour; and attention to information about seasonal influenza vaccination) were confirmed as the final solution. The predictor importance of these four segmentation variables ranged from 0.76 to 1.00, confirming that they are highly important to the cluster formation [28].

**Figure 1:**
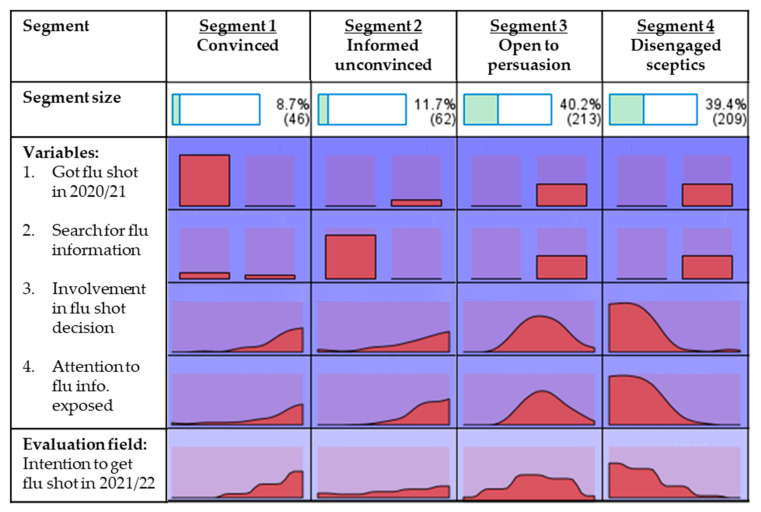
Distribution of segmentation variables by segment.

The most important variable for cluster formation in this study is the behaviour of being vaccinated in the current season (2021–2021) (importance = 1.00). The behaviour of actively searching for information about seasonal flu (importance = 0.88) and the level of involvement in influenza vaccination decisions (importance = 0.84) were highly important to cluster formation. Moreover, when exposed to information or news about seasonal influenza, the level of attention paid to the information was also relatively important to cluster formation (importance = 0.76). To validate the segment solutions, chi-square tests were conducted on the two categorical variables (i.e., influenza behaviour and information search) and ANOVA tests on the two continuous variables (i.e., personal involvement and attention to information). Significant statistical differences (*p* < 0.001) were observed between segments for all variables included. A summary of the two-step cluster analysis results is provided in Table 2. The four clusters representing different degrees of vaccination behaviour, level of involvement in making vaccination decision, active information search, and attention to seasonal influenza vaccination are delineated below. Moreover, sociodemographic, psychographics, and behavioural characteristics of identified segments are examined by drawing on the research data collected. The distribution among the four clusters was unequal, with sizes of 9%, 12%, 40%, and 39%. Due to unequal sample size, the assumption of homogeneity of variances was violated in one-way ANOVA tests for all continuous variables as Levene’s tests were significant. Therefore, Welch ANOVA tests were conducted in this study instead of the standard one-way ANOVA. Due to the heterogeneity of variances, Games-Howell tests were conducted as post hoc tests to identify segment differences. Figure 1 shows the graphical distribution of segmentation variables by segment.

### 3.1. Cluster Delineation

#### 3.1.1. Segment One: High Involvement, Convinced

This segment is dominated by individuals who had received seasonal influenza vaccines in the last season. All students in this segment were vaccinated in the 2020–2021 influenza season as compared to 0% in all other segments. While 100% of students in this segment were vaccinated, most (80%) do not actively search for seasonal influenza information. This segment is also distinguished from other segments by their high level of involvement in making seasonal influenza vaccination decisions (*M* = 5.41, *p* < 0.001). Moreover, this segment of students tends to pay greater attention to seasonal influenza information exposed to them through various media and communication channels (*M* = 4.61, *p* < 0.001). This is the smallest segment with a cluster size of 9% (*n* = 46).

#### 3.1.2. Segment Two: High Involvement, Informed Unconvinced

This segment comprises individuals who had not received seasonal influenza vaccines in the last season. None of the students (0%) in this segment were vaccinated in the 2020–2021 influenza season. Although students in this segment did not get vaccinated, the segment is distinguished from other non-vaccinated segments by the students’ interest in influenza-related information. All students in this segment (100%) actively search for seasonal influenza information, and their attention to the influenza-related information exposed to them is the highest among all segments (*M* = 5.26, *p* < 0.001). Hence, this segment is also highly involved in making seasonal influenza vaccination decisions (*M* = 4.73, *p* < 0.001). This is the second smallest segment with a cluster size of 12% (*n* = 62).

#### 3.1.3. Segment Three: Low Involvement, Open to Persuasion

This is the largest segment with a cluster size of 40% (*n* = 213). Similar to segment two, none of the individuals in this segment (0%) had received seasonal influenza vaccines in the last season. Unlike segments one and two, students in this segment have a low level of involvement in making seasonal influenza vaccination decisions (*M* = 4.37, *p* < 0.001), and none of the students (0%) actively search for seasonal influenza information. However, when exposed to seasonal influenza information through media, social, or mass communication channels, students in this segment pay a high level of attention to the information (*M* = 4.54, *p* < 0.001).

#### 3.1.4. Segment Four: Low Involvement, Disengaged Sceptics

This segment is almost as big as the largest segment (cluster size of 39%, *n* = 209). Like segments two and three, this segment is also a non-vaccinated group. None of the individuals (0%) in this segment had received a seasonal influenza vaccination in the 2020–2021 season. This segment is distinguished from other segments by their significantly low level of involvement in making seasonal influenza vaccination decisions (*M* = 2.40, *p* < 0.001). Moreover, none of the students (0%) actively search for seasonal influenza information, and they pay minimal attention to seasonal influenza information exposed through any communication channels (*M* = 2.70, *p* < 0.001).

### 3.2. Segment Characteristics—Sociodemographic and Health Conditions

When segmenting the general public using sociodemographic variables, significant differences were observed between groups along the vaccine hesitancy continuum [15]. However, in this study (see Table 3), sociodemographic differences between segments of university students were not significant in terms of sex (*p* = 0.986), year of study (*p* = 0.332), or academic results (*p* = 0.395).

The findings reveal that academic performance does not relate to the perceived need for influenza vaccination to prevent influenza-like illnesses that may affect academic results. The only factor that provided a statistically significant difference among segments is the area of study (*p* = 0.047). The vaccinated segment (segment one) has a significantly higher proportion of students from social sciences and health-related programmes whereas over one-third of respondents from business programmes appear in the non-vaccinated segments. The results indicate that students who major in programmes leading to contact with vulnerable people in internships or careers upon graduation tend to receive the influenza vaccine.

Considering factors related to students’ family circumstances (see Table 4), there is no statistical difference between segments based on whether students are living with or in frequent contact with a high-risk population including elderly people (*p* = 0.545), young children (*p* = 0.299), or people with chronic illness (*p* = 0.053). 

However, segment one (vaccinated segment) differs significantly from other segments (non-vaccinated segments) in terms of the number of family members who received the influenza vaccine in the 2020–2021 season as determined by Welch ANOVA (*F*(3, 135.484) = 75.074, *p* < 0.001). For seasonal influenza vaccination behaviour of family members, 28% of segment one students have all family members and 48% have more than half of family members vaccinated whereas the percentages in the other segments are less than 2% and 5–10% respectively. Only 2% of segment one students come from a family in which no one received an influenza vaccination in the last season but the percentages for other segments are 74% (segment two), 78% (segment three), and 85% (segment four). 

Segment characteristics in terms of personal health conditions are presented in Table 4. There is no group difference between segments based on personal health conditions in terms of whether the students had received other vaccines voluntarily (*p* = 0.073), had a severe allergic reaction to vaccine injection (*p* = 0.305), and frequency of flu-related illness in the past five years (*p* = 0.773). However, segment one (vaccinated group) is significantly different from other segments (non-vaccinated) in terms of physical health condition (26% of segment one students have chronic diseases vs around 10% in other segments, *p* = 0.007).

### 3.3. Segment Characteristics—Influenza Vaccine-Related Behaviours, Attitudes, and Intentions

Seasonal influenza vaccination behaviours, attitudes, and intentions are key in the differentiation between segments as all factors have a statistically significant difference in chi-square or ANOVA tests (see Table 5).

All individuals (100%) in segment one were vaccinated in the 2020–2021 season but none of the students (0%) in all other segments received seasonal influenza vaccine (*p* < 0.001). For frequency of influenza vaccination in the past five years, only 2% of segment one did not receive an influenza vaccine, but a substantial proportion of other segments did not (segment two—85%, segment three—74%, segment four—94%, *p* < 0.001). While 37% of students in segment one were vaccinated every year in the last five years, only 2% of segment two and none (0%) of segments three and four were vaccinated. Moreover, 24% of segment one had vaccinated for three to four years and 37% for one to two years compared to 2% and 11% for segment two, 5% and 21% for segment three, and 0% and 6% for segment four.

Attitudes towards seasonal influenza vaccination measured using the 5Cs model also revealed a statistically significant difference between groups as determined by Welch ANOVA for confidence (*F*(3, 141.093) = 24.862, *p* < 0.001), complacency (*F*(3, 140.233) = 61.703, *p* < 0.001), convenience (*F*(3, 136.856) = 7.522, *p* < 0.001), calculation (*F*(3, 137.244) = 3.247, *p* = 0.024), and collective responsibility (*F*(3, 138.103) = 8.864, *p* < 0.001). A Games-Howell post-hoc test revealed that the convinced segment (segment one) has significantly higher confidence in seasonal influenza vaccines (*M* = 5.12 ± 0.82, *p* < 0.001) than all other segments. The informed unconvinced segment (segment two) has the lowest level of confidence (*M* = 3.74 ± 1.11) and has no statistically significant difference compared to the disengaged sceptics segment (segment four) (*M* = 4.02 ± 1.05, *p* = 0.303). Moreover, segment two has significantly lower confidence in influenza vaccines than the open to persuasion segment (segment three) (*M* = 4.16 ± 0.76, *p* = 0.035). For complacency, segment one (the convinced) has statistically significant lower complacency (i.e., higher perceived risks) of influenza-like illness preventable by influenza vaccine (*M* = 2.80 ± 0.93, *p* < 0.001) whereas segment four (the disengaged sceptics) has the highest complacency (*M* = 4.62 ± 0.90, *p* < 0.001) compared to all other segments. There was no statistically significant difference between segments two (*M* = 3.54 ± 1.03) and three (*M* = 3.91 ± 0.85, *p* = 0.056). For convenience, the convinced (segment one) perceived that receiving an influenza vaccine was convenient and the perceived convenience was significantly higher than all other segments (*M* = 4.57 ± 1.21, *p* < 0.001). There was no statistically significant difference between the other three groups; all these segments did not perceive getting the influenza vaccination as convenient (*M_segment 2_* = 4.04 ± 0.94; *M_segment 3_* = 3.78 ± 0.78, *p* = 0.196; *M_segment 4_* = 3.73 ± 1.00, *p* = 0.125).

For calculation, the disengaged sceptics (segment four) has a statistically significant lower effort in evaluating the risks and benefits of influenza vaccination than all other segments (*M* = 4.63 ± 1.11, *p* = 0.024). There was no statistically significant difference between the other three groups and all other segments make vaccination decisions based on risk and benefit calculation (*M_segment 1_* = 4.80± 1.38; *M_segment 2_* = 4.95 ± 1.10, *p* = 0.932; *M_segment 3_* = 4.93 ± 0.94, *p* = 0.936). For attitude towards vaccination as a collective responsibility in society, the disengaged sceptics (segment four) and the informed unconvinced (segment 2) were significantly weaker compared to the other segments (*M_segment 4_* = 4.27± 1.25; *M_segment 2_* = 4.65 ± 1.26, *p* = 0.100; *M_segment 1_* = 4.98 ± 1.32, *p* < 0.001; *M_segment 3_* = 4.79 ± 0.94, *p* < 0.001). 

Finally, the intention to be vaccinated in the upcoming (2021–2022) season was examined (*F*(3, 143.269) = 106.193, *p* < 0.001). A Games-Howell post-hoc test revealed that the intention to be vaccinated was significantly higher among the convinced (segment one) (*M* = 5.27 ± 1.14, *p* < 0.001) compared to other segments. The disengaged sceptics (segment 4) have significantly lower intention to be vaccinated (*M* = 2.17 ± 1.22, *p* < 0.001) compared to the informed unconvinced segment (*M* = 3.66 ± 1.81) and the open to persuasion segment (*M* = 3.63 ± 1.47). There was no statistically significant difference between the informed unconvinced (segment two) and open to persuasion (segment three) groups (*p* = 0.998). Mean scores of segments two, three, and four were lower than 4.00 on the 7-point Likert scale, indicating that they generally do not intend to receive a seasonal influenza vaccination in the coming 2021–2022 season.

### 3.4. Segment Characteristics—Influenza-Related Information Search, Exposure, and Trust

The data presented in Table 6 reveal that only segment one (20% of the convinced) and segment two (100% of the informed unconvinced) actively search for information about seasonal influenza. 

None of the students (0%) from segments three (the open to persuasion) and four (the disengaged sceptics) actively search for influenza-related information from any sources. The informed unconvinced (segment two) reported that they usually search for influenza-related information through search engines (97%), family members or friends (87%), social media sites (86%), and online forums (71%). All students in this segment actively search for influenza-related information and they heavily rely on online platforms including search engines, social media sites, and online forums. The informed unconvinced segment has strong trust in these information sources (mean = 5.23) although it is reported that most misinformation in these online sources significantly influence the biasness towards vaccinations [29]. Thus, although only students in this segment (informed unconvinced) actively search for influenza information and they pay the highest level of attention to the information exposed (mean = 5.26), their confidence in influenza vaccines (mean = 3.47) is lowest among all respondents (see Table 5).

Among the 20% of the convinced (segment one) who reported having actively searched for influenza-related information, their most-used channels were search engines (100%), family members or friends (78%), and government websites (67%). Only 56% of students from segments one and two search for influenza information from doctors or other health professionals. The level of trust in usual influenza information search channels was relatively high (*M_segment 1_* =5.22; *M_segment 2_* = 5.23) and there is no statistically significant difference between these two segments (*p* = 0.992). 

**Figure 2:**
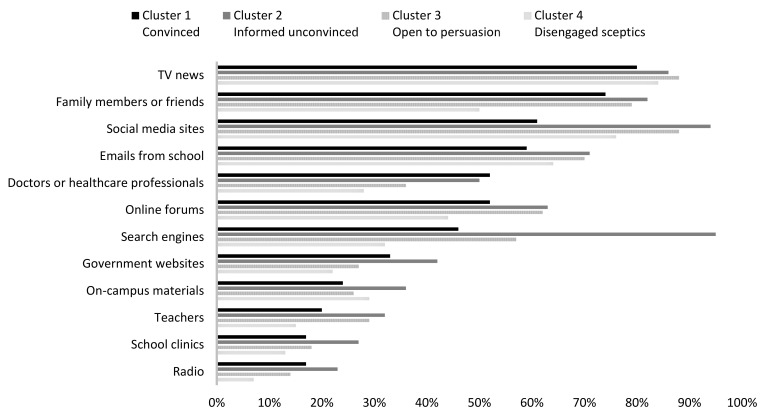
Sources from which most flu-related information was received by segment.

When exposed to seasonal influenza-related information, there was a statistically significant difference between segments in terms of attention to the information exposed, as determined by Welch ANOVA (*F*(3, 131.264) = 106.193, *p* < 0.001). The disengaged sceptics (segment four) significantly showed low interest in influenza-related information when exposed (*M* = 2.70 ± 1.05, *p* < 0.001) and the informed unconvinced (segment two) reported significantly high interest (*M* = 5.26 ± 0.90) compared to the convinced segment (*M* = 4.61 ± 1.42, *p* = 0.041) and the open to persuasion segment (*M* = 4.54 ± 0.64, *p* < 0.001). Figure 2 compares the sources from which most flu-related information was received by segment.

The top sources from which most influenza-related information was received by all segments include TV news (*f*_segment one_: 80%; *f*_segment two_: 86%; *f*_segment three_: 88%; *f*_segment four_: 84%; *p* = 0.465), social media sites (*f*_segment one_: 61%; *f*_segment two_: 94%; *f*_segment three_: 88%; *f*_segment four_: 76%, *p* < 0.001), emails from school (*f*_segment one_: 59%; *f*_segment two_: 71%, *f*_segment three_: 70%; *f*_segment four_: 64%, *p* = 0.362), and family members or friends (*f*_segment one_: 74%; *f*_segment two_: 82%; *f*_segment three_: 79%; *f*_segment four_: 50%, *p* < 0.001). Search engines were reported as the most frequent sources from which the informed unconvinced (segment two) were exposed to influenza-related information but were not among the top five sources for other segments (*f*_segment one_: 46%; *f*_segment two_: 95%; *f*_segment three_: 57%; *f*_segment four_: 32%, *p* < 0.001). A large proportion of students in segments two (the informed unconvinced) and three (the open to persuasion) reported that online forums were major channels through which they were exposed to influenza-related information but this is not the case for segments one (the convinced) or four (the disengaged sceptics) (*f*_segment one_: 52%; *f*_segment two_: 63%; *f*_segment three_: 62%; *f*_segment four_: 44%, *p* < 0.001). 

Although students from all segments received most influenza-related information from similar channels, the level of trust in these sources is statistically significantly different (*F*(3, 144.169) = 8.162, *p* < 0.001). The disengaged sceptics (segment four) reported significantly lower trust in information channels from which they were exposed to influenza information (*M* = 4.63 ± 1.14, *p* < 0.001) compared to all other segments. However, there was no statistically significant difference between other three segments (*M_segment 1_* = 5.20 ± 0.80; *M_segment 2_* = 5.12 ± 0.82, *p* = 0.973; *M_segment 3_* = 4.99 ± 0.71, *p* = 0.493).

### 3.5. Segment Characteristics—Flu Vaccination Promotion Influencer and Message Concept Testing

**Figure 3:**
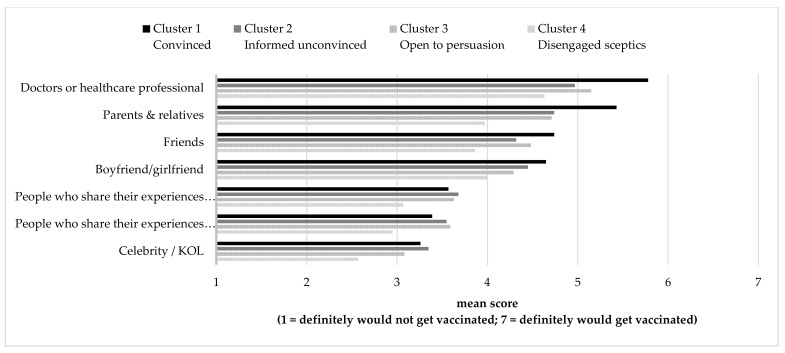
Claimed effectiveness of influencer in motivating seasonal influenza by segment.

**Figure 4:**
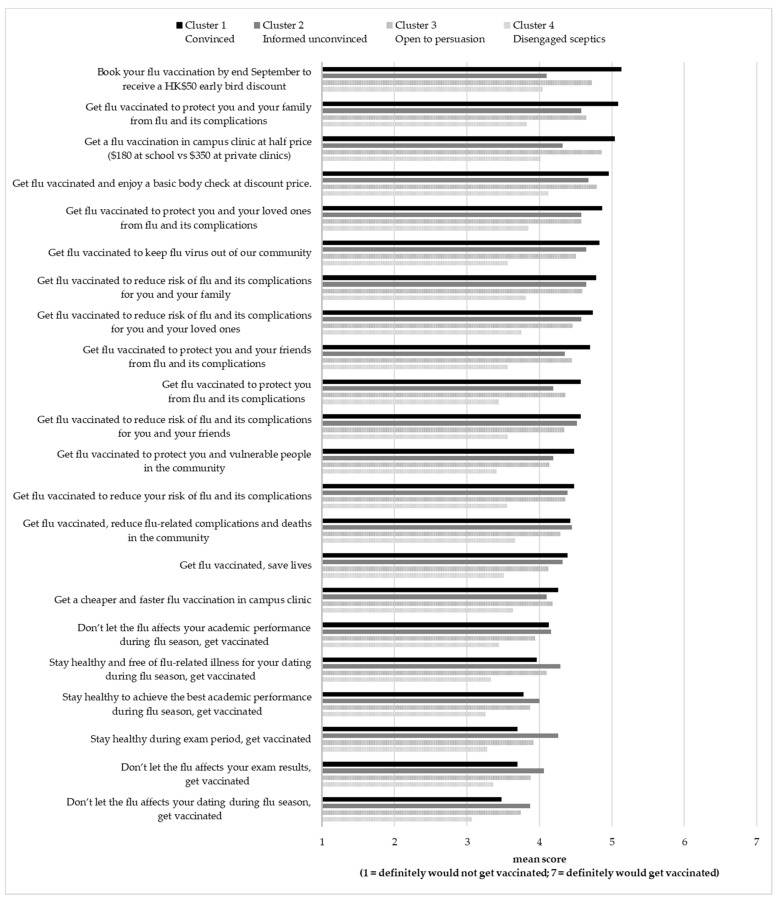
Claimed effectiveness of promotion taglines in motivating seasonal influenza by segment.

The survey also examined students’ perception of the effectiveness of vaccination promotion messages and influencers as a concept test of ideas generated in focus group studies prior to this survey. A total of 7 influencer types (see Figure 3) and 22 message taglines (see Table 7 and Figure 4) were examined. In terms of influencers (Figure 3), doctors or healthcare professionals were reported as the only effective influencers for the disengaged sceptics (segment four: *M* = 4.63), and all other influencers were not influential (*M* < 4.0). For all other segments, doctors or healthcare professionals were found to be the most effective influencers (*M_segment 1_* = 5.78; *M_segment 2_* = 4.97; *M_segment 3_* = 5.15) followed by parents and relatives (*M_segment 1_* = 5.43; *M_segment 2_* = 4.74; *M_segment 3_* = 4.71).

Results for the 22 message taglines (Figure 4) were grouped into six types of messages including the message focus (promotion of benefit, prevention of risk, vs incentive to act), and orientation (individual-oriented, collective-oriented family and friends, vs collective-oriented community), to derive a better understanding of the message strategy (Table 7). 

Mean scores of the likelihood to be motivated to receive seasonal influenza vaccination rated by segment four (the disengaged sceptics) were about or below 4 on a 7-point Likert scale for all promotion messages, indicating that this segment will not be convinced to receive an influenza vaccine regardless of promotion messages. Incentive focus messages including ‘get a flu vaccination in campus clinic at half price ($180 at school vs $350 at private clinics)’, ‘book your flu vaccination by end September to receive a HK$50 early bird discount’, and ‘get flu vaccinated and enjoy a basic body check at discount price’, were deemed to be the most promising promotion message focus to motivate segments one (the convinced: *M* = 4.85) and three (the open to persuasion: *M* = 4.64) to get vaccinated. Moreover, collective-oriented (family and friends) messages were also reported as effective in motivating all segments (except segment four, the disengaged sceptics) to be vaccinated (*M_segment 1_* = 4.79; *M_segment 2_* = 4.54; *M_segment 3_* = 4.51, *p* < 0.001). 

The collective-oriented (family and friends) messages appeared to work equally well no matter how they were framed in the promotion focus: ‘get flu vaccinated to protect you and your family from flu and its complications’, and ‘get flu vaccinated to protect you and your loved ones from flu and its complications’ or prevention focus ‘get flu vaccinated to reduce risk of flu and its complications for you and your family’ and ‘get flu vaccinated to reduce risk of flu and its complications for you and your loved ones’. Messages that talked about vaccination and better health conditions for study, examinations (usually during flu season), and dating were all reported to be ineffective (mean scores below 4.0 for all segments, *p* < 0.001). 

### 3.6. Segment Characteristics—COVID-19 Vaccine-Related Behaviours, Attitudes, and Intentions

The differentiation between segments in terms of COVID-19 vaccine-related behaviours, attitudes (except for complacency), and intentions produced statistically significant differences in chi-square and ANOVA tests (see Table 8). In terms of COVID-19 vaccination behaviour, the seasonal influenza-vaccinated segment (segment one) has a significantly higher proportion of individuals who were COVID-19 vaccinated (segment one—30%; segment two—13%; segment three—6%; segment four—8%, *p* < 0.001). Family members of those in segment one also have a higher COVID-19 vaccination rate compared to the other segments. For 35% of segment one, none of their family members have received a COVID-19 vaccine, but over two-thirds of the other segments have no one vaccinated in the whole family (segment two—63%; segment three—64%; segment four—67%, *p* < 0.001). In terms of COVID-19 vaccination attitude, there was a statistically significant difference between segments in the level of involvement in making a decision to be vaccinated (*F*(3, 149.544) = 14.782, *p* < 0.001).

Segment four (the disengaged sceptics) has a statistically significant lower level of involvement in COVID-19 vaccination decisions (*M* = 4.19 ± 1.82, *p* < 0.001) compared to other segments (*M_segment 1_* = 5.18 ± 1.45; *M_segment 2_* = 5.38 ± 1.01; *M_segment 3_* = 4.91 ± 1.27). Attitudes towards COVID-19 vaccination measured using the 5Cs model demonstrate a statistically significant difference between segments as determined by Welch ANOVA for confidence (*F*(3, 143.657) = 9.692, *p* < 0.001), convenience (*F*(3, 143.590) = 5.318, *p* = 0.002), calculation (*F*(3, 149.172) = 3.577, *p* = 0.015), and collective responsibility (*F*(3, 145.051) = 6.259, *p* < 0.001). However, there was no statistically significant difference between segments for complacency (*F*(3, 141.766) = 1.110, *p* = 0.347). With mean scores below 4.0, all segments reported low confidence in COVID-19 vaccines (*M_segment 1_* = 3.15 ± 1.04; *M_segment 2_* = 3.19 ± 1.21; *M_segment 3_* = 3.19 ± 1.05; *M_segment 4_* = 2.64 ± 1.17) and low complacency (*M*_segment 1_ = 3.12 ± 1.06; *M*_segment 2_ = 3.17 ± 1.23; *M*_segment 3_ = 3.38 ± 0.99; *M*_segment 4_ = 3.30 ± 1.19). While segment four has no opinion regarding the convenience of receiving COVID-19 vaccines (*M* = 4.09 ± 1.55), a statistically significant difference was found compared to segment two (*M* = 4.85 ± 1.27) but not with segment one (*M* = 4.50 ± 1.66, *p* = 0.424) or segment three (*M* = 4.36 ± 1.36, *p* = 0.227). All segments indicated a medium to high level of calculation in considering the risks and benefits of COVID-19 vaccine uptake and a statistically significant difference was observed between segments two and four only (M_segment 2_ = 4.73 ± 1.13; M_segment 4_ = 4.27 ± 1.35, *p* = 0.040; M_segment 1_ = 4.71 ± 1.08, *p* = 1.00; M_segment 3_ = 4.55 ± 1.18, *p* = 0.705). For receiving COVID-19 vaccines as a collective responsibility, segment four indicated that they were unlikely to get vaccinated to protect others in the community (*M* = 3.82), whereas all other segments reported similar levels of willingness to uptake COVID-19 vaccines (M_segment 1_ = 4.44 ± 1.55; M_segment 2_ = 4.59 ± 1.35, *p* = 0.950; M_segment 3_ = 4.33 ± 1.40, *p* = 0.972). The intention to receive COVID-19 vaccination was generally low among the respondents, with segment four significantly lower than the other segments (M_segment 1_ = 4.10 ± 2.01; *M_segment 2_* = 3.95 ± 1.82; *M_segment 3_* = 3.98 ± 1.78; *M_segment 4_* = 3.12 ± 2.00, *p* < 0.001).

**Figure 5:**
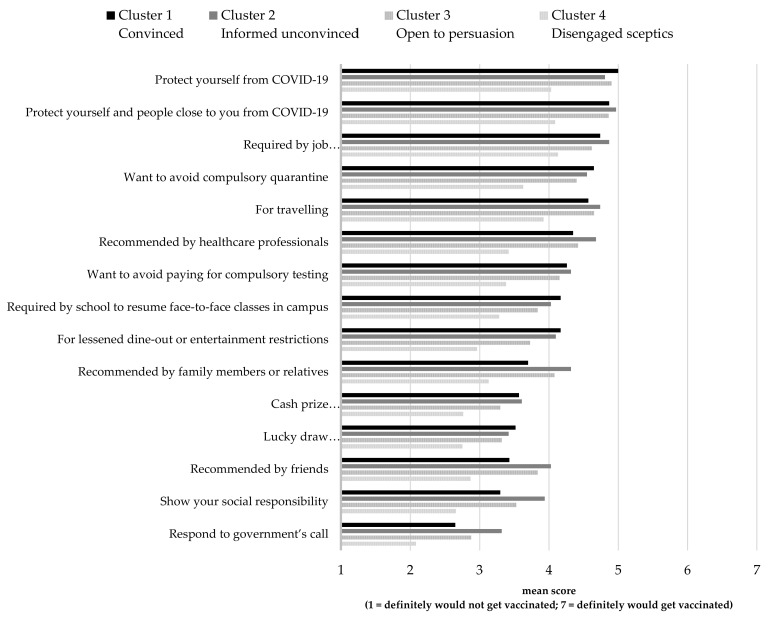
Reasons that may motivate COVID-19 vaccination by segment.

Students were also asked to rate how likely they were to receive COVID-19 vaccination because of existing reasons including: (i) health protection (recommended by health professionals); (ii) travel and entertainment (required by the government); (iii) fulfilling internship or job requirements (required by the government); (iv) social responsibility or herd immunity (recommended by health professionals); (v) fulfilling resumption of face-to-face teaching (required by school); (vi) social reasons (recommended by family members or friends); and (vii) incentives (prizes offered by various organisations or commercial companies). Rating of their likelihood to be motivated to vaccinate for these reasons is shown in Figure 5. 

With mean scores lower than 4.0, the disengaged sceptics (segment four) indicated that they are unlikely to receive COVID-19 vaccines for any of the above reasons. Other segments shared a similar level of willingness to receive COVID-19 vaccines with responses to three most important reasons: *‘protect yourself from COVID-19′* (M_segment 1_ = 5.00 ± 1.89; M_segment 2_ = 4.81 ± 1.57, *p* = 0.342; M_segment 3_ = 4.90 ± 1.53, *p* = 0.985); *‘protect yourself and people close to you from COVID-19′* (M_segment 1_ = 4.87 ± 1.76; M_segment 2_ = 4.97 ± 1.52, *p* = 0.990; M_segment 3_ = 4.86 ± 1.47, *p* = 1.000); and *‘required by job (e.g., internship, part-time or full-time job)’* (M_segment 1_ = 4.74 ± 1.82; M_segment 2_ = 4.87 ± 1.35, *p* = 0.976; M_segment 3_ = 4.62 ± 1.69, *p* = 0.979). Next, as travelling and other overseas activities such as exchange programmes are suspended because of COVID-19, students (except those from segment four) also reported that they would vaccinate for the sake of ‘*travelling*’ (M_segment 1_ = 4.57 ± 2.11; M_segment 2_ = 4.74 ± 1.72, *p* = 0.966; M_segment 3_ = 4.65 ± 1.85, *p* = 0.994) and ‘*avoidance of compulsory quarantine*’ (M_segment 1_ = 4.65 ± 1.95; M_segment 2_ = 4.55 ± 1.72, *p* = 0.992; M_segment 3_ = 4.40 ± 1.80, *p* = 0.849). Reasons including incentives (e.g., lucky draw and instant prize for everyone), social responsibility, and resumption of face-to-face classes were not motivating factors, with mean scores below 4.0 for all segments.

## 4. Discussion

This study identified four homogeneous segments of students by using level of involvement in influenza vaccination decisions, information seeking and attention to influenza-related information, and influenza vaccination behaviours. The study suggests a new approach to profiling health campaign audiences to inform the development of tailored vaccination promotion and communication programmes in tertiary education institutions. Four distinctive segments that differ across their area of study, personal and family vaccination behaviour, attitudes and intentions towards seasonal influenza vaccination, influenza-related information source channels, and responses to vaccine promotion messages and influencers were identified. The segment size also reflects the current norm that a significantly large portion of healthy young adults do not take influenza vaccines and do not perceive that seasonal influenza is an illness to be prevented by vaccination.

The ‘convinced’ segment is the vaccinated group as almost all of them received seasonal influenza vaccination more than once in the last five years. Moreover, almost all of them have family members who were vaccinated during the last influenza season. Almost half of the segments comprise students studying social sciences or health-related programmes. Although influenza vaccination is a high involvement decision to them, only 20% of the segment actively search for information, which indicates that they possibly act on pre-existing knowledge and attitude towards influenza vaccination as they have high confidence in the influenza vaccine and a high perceived risk of influenza-related disease that is preventable by vaccine. Moreover, being individually responsible for protecting others from influenza-like illness is a major reason for their vaccination behaviour. They also have a high intention to be vaccinated in the coming (2021–2022) seasonal influenza season. Among all promotional messages tested, this segment rated ‘early bird discount’ as the most effective message, followed by ‘vaccinated to protect oneself and family’. Therefore, a promotion campaign targeting this segment of students does not need to provide detailed information about why seasonal influenza vaccination is important. The campaign should motivate the audiences to act by providing information related to campaign logistics (e.g., when, where, and how to register) and the incentives (e.g., early bird discount) of the vaccination programme. Moreover, vaccination commitment from this segment could be secured before the influenza season by using early bird discounts. This group could be reached by a school email sent specifically to students who had been vaccinated at the school health clinic in previous years, students studying social sciences and health-related programmes, or those who have responded to the early bird promotion. As most of their family members are also receptive to influenza vaccination, school clinics may also consider package promotion for students to receive a vaccination with their families. This segment should be recruited to champion vaccine uptake given that they have full confidence in flu vaccination.

The ‘informed unconvinced’ segment mainly comprises individuals (85%) who have not been vaccinated in the last five years. All of them did not receive a seasonal influenza vaccination and 74% have no one from their family vaccinated in the 2020–2021 season. This segment is highly involved in making influenza vaccination decisions and has relatively stronger beliefs that influenza-like illness has a high risk to health that is preventable by vaccination. When exposed to influenza-related information, this segment pays more attention to the news or information compared to respondents from other segments. Their high level of attention to information related to seasonal influenza is also demonstrated by their information-seeking behaviour. However, although 100% actively search for influenza-related information, they have the lowest confidence in influenza vaccines. Thus, their intention to vaccinate in the coming seasonal influenza season is low. Over 95% of individuals in this segment search for or are exposed to influenza-related information mainly from search engines, online forums, social media sites, and family members or friends. This segment reported a high level of trust in information obtained from these channels and appeared to have strong, pre-determined attitudes that the seasonal influenza vaccine is not safe, effective, or may cause strong side-effects. Online information from discussion forums and social media may be delivering misinformation contributing to lower confidence in flu vaccines [29,30]. Therefore, the need for fact-checking tools across search engines and social media sites is advocated to fight misinformation that can significantly influence the biasness towards vaccinations [31,32,33]. Moreover, approaches involving champions are likely to encourage the informed unconvinced segment to receive their flu vaccination given they trust family and friends.

The ‘open to persuasion’ segment also consists of a large portion of individuals (74%) who have not been vaccinated in the last five years. All of them did not receive a seasonal influenza vaccination, but 25% have family members who were vaccinated in the 2020–2021 season. This segment does not actively search for influenza-related information because seasonal influenza vaccination is a low involvement decision for them. Although they do not actively search for information, they do pay attention to influenza-related information or news when exposed to it. They do not hold a strong view about whether seasonal influenza vaccines are safe and effective (confidence) or necessary to prevent risks from influenza-like illness (complacency). Among all promotional messages in the concept testing, this segment provided the highest rating to incentive focus messages including ‘get vaccinated at campus clinic at half price’, ‘early bird discount’, and ‘enjoy a basic body check at discount price’, followed by ‘vaccinated to protect oneself and family’. As this segment does not have a strong pre-disposition regarding the efficacy and necessity of seasonal influenza vaccination and reported they can be persuaded by incentives, a vaccination promotion campaign targeting this segment should aim to draw their attention and attract them to act. At least 70% of individuals in this segment pay attention to influenza-related information received via emails from school. Since students receive many emails from school covering aspects such as academic, scholarship, internship, and school activities every day, the vaccination promotion emails should have an enticing subject to encourage this segment to open the emails.

The ‘disengaged sceptics’ segment is the most difficult segment to be persuaded. None of the individuals in this segment were influenza vaccinated in the 2020–2021 season and only 6% received a vaccination once or twice in the last five years. For the upcoming seasonal influenza season (2021–2022), 98% of this segment indicated they would not or were unlikely to be vaccinated. They neither actively search for influenza-related information nor pay attention to related news or information when exposed to it. Although they do not have a strong view of confidence in influenza vaccines, they do not believe that influenza-like disease causes serious illness that needs to be prevented by vaccination. Although they claimed that doctors or healthcare professionals are effective influencers to promote seasonal influenza vaccination, they did not report that any of the messages tested in the concept testing were effective in motiving them to vaccinate. Thus, as a group, this segment is considered the hardest to reach. Promotional efforts should be directed to the first three segments, and over time as flu vaccination is normalised across segments 2 and 3, changes in segment 4′s openness to consider flu vaccination will become evident.

The findings of this study demonstrate how audience segmentation can be applied to inform the design of effective vaccination campaigns that can be delivered according to distinct segment characteristics. As a core social marketing principle, audience segmentation delivers a guide demonstrating how prioritisation of target audiences can occur and how social marketing campaigns can be tailored to distinct student groups on campus to drive vaccine uptake. The focus of campaigns could range from securing the 9% vaccinated and ‘convinced’ segment to revaccinate this season to nudging the 40% ‘open to persuasion’ segment to vaccinate. Achieving a 30% vaccination rate on campus will significantly contribute to the herd immunity target.

In addition, COVID-19 vaccination could be cross-promoted to students in the seasonal influenza vaccination campaign. The ‘convinced’ segment (segment one) has the highest positive intention to receive COVID-19 vaccination. Thus, this group of students is easily reached by emails using last year’s seasonal influenza vaccination records. School clinics may send emails to these students and explain the benefits of receiving both seasonal influenza and COVID-19 vaccines. Incentives could be offered to students who receive both seasonal influenza and COVID-19 vaccines. Students may also be given health advice in terms of the vaccination schedule by doctors at school clinics. Moreover, COVID-19 vaccination can be cross-promoted to these students through their family members. According to the findings, 98% of the ‘convinced’ segment have family members who received seasonal influenza vaccines and 65% for COVID-19 vaccines (see Table 5 and Table 8). Family is the second most important information channel and vaccination-related influencer (see Figure 3 and Figure 4 and Table 6). For example, family packages could be offered to motivate those who receive seasonal influenza or COVID-19 vaccines to influence their family members to get vaccinated. Segment two and three prospects could be identified and the COVID-19 vaccines cross-promoted through school clinics when they respond to the 2021–2022 seasonal influenza vaccination promotional activities. According to the survey results, segment four, who are uninterested and sceptical about seasonal influenza vaccinations, are also exceedingly resistant to COVID-19 vaccines. In time approaches that report vaccination rates across different student groups can be used to encourage this hesitant group to vaccinate.

## 5. Implications and Conclusions

Currently, there is only limited research that focuses on promoting seasonal influenza vaccination among healthy young adults such as university students. This study is a response to the WHO [14] call to segment the target audiences for the development of effective social marketing campaigns to motivate vaccination for seasonal influenza and now for COVID-19. Audience segmentation is a basic benchmark of social marketing campaign development. This study highlights important managerial implications for healthcare policy makers and senior management in tertiary education institutions. It also offers insights into the setting of realistic vaccination targets as achieving a high vaccination rate among university students may be unrealistic. According to the findings, at least 40% of students expressed that they would not uptake both seasonal influenza and COVID-19 vaccination. However, this study identified determinants that extend the ability of healthcare professionals to segment the student market more effectively. The findings also present important insights for designing vaccination programmes targeting students who are highly likely to be motivated to repeat their seasonal influenza vaccination annually or receive their first vaccination if a tailored promotion strategy is applied. Many public health promotion programmes focus on health education and risk communication. However, this study identified student segments that are persuadable, convinced individuals and students with no strong pre-determined attitudes towards vaccination. Messages that can be applied to motivate emphasise incentives. Future seasonal influenza vaccination campaigns may test the effectiveness of messages highlighting protection for oneself and family and cost savings of vaccines administered by school clinics. Early bird discounts and other promotional tactics should be offered to secure vaccination intention and commitment, in turn creating champions who may convince others to roll up their sleeves to become vaccinated.

The study has two major theoretical contributions. First, segmentation is a key component in the formulation of social marketing programmes. With limited research exploring the potential value of social marketing in vaccination campaigns, this study provides a more comprehensive profile of university student segments by considering a wide range of variables related to students’ sociodemographic, psychographic, and behavioural attributes as a reference for future social marketing research. The audience segments identified in this study will also facilitate multi-group analysis for future empirical research examining theoretical health communication or promotion frameworks. As distinct audience segments respond differently to various marketing, communication, psychological, or behavioural variables, running a multi-group analysis using these pre-defined segments will enrich the research outcomes.

This study has several limitations. Although the sample was representatively collected from a population of 11,000 students, it was collected from a single university in Hong Kong. Further studies should be expanded to more universities in Hong Kong, as well as other countries, to enhance the generalisability of the findings. Moreover, this study did not attempt to explain behaviour; it only examined how different segments respond to messages in the concept testing. Further research should be conducted to examine the applicability and usefulness of the segments identified in this audience segmentation aiming to promote uptake of seasonal influenza vaccines and possibly COVID-19 vaccines among university students. This study provides some insights into a range of motivations identifying segment differences. A limitation of this study is that it did not directly test different price points for the COVID-19 vaccines. Future research is recommended to examine choice preferences. Techniques such as Best Worst scaling can be used to accurately identify pricing preferences.

This study provides audience segmentation analysis demonstrating how cluster analysis can be applied to usefully unpack heterogeneity among university students gaining insights into attitudinal and behavioural determinants in seasonal influenza vaccination uptake (or not) and influenza-related information seeking. Moreover, the study offers new theoretical directions to investigate healthy young adults as most extant influenza research, and promotion campaigns focus primarily on high-risk populations and healthcare workers. Thus, this study provides practical methods to understand audience segments aiming to inform the design of effective social marketing campaigns for motivating seasonal influenza vaccination and cross-promoting COVID-19 vaccines among university students.

## Figures and Tables

**Table 1:** Auto-clustering statistics.

Number of Clusters	Schwarz’s Bayesian Criterion (BIC)	BIC Change ^a^	Ratio of BIC Changes ^b^	Ratio of Distance Measures ^c^
1	1501.610			
2	995.011	−506.599	1.000	2.177
3	782.706	−212.305	0.419	1.061
4	584.765	−197.942	0.391	3.606
5	557.064	−27.700	0.055	1.318
6	545.124	−11.940	0.024	1.083
7	536.970	−8.154	0.016	1.582
8	545.671	8.700	−0.017	1.132
9	557.751	12.080	−0.024	1.672
10	580.099	22.348	−0.044	1.096
11	603.787	23.688	−0.047	1.008
12	627.585	23.798	−0.047	1.024
13	651.712	24.127	−0.048	1.059
14	676.597	24.885	−0.049	1.223
15	703.806	27.209	−0.054	1.204

^a.^ The changes are from the previous number of clusters in the table. ^b^ The ratios of changes are relative to the change for the two cluster solution. ^c^ The ratios of distance measures are based on the current number of clusters against the previous number of clusters.

**Table 2:** Summary of two-step cluster analysis results (*n* = 530).

Segmentation Variable	Importance		Segment 1 Convinced	Segment 2 Informed Unconvinced	Segment 3 Open to Persuasion	Segmemnt 4 Disengaged Sceptics	Chi Square/ANOVA Test (*p*-Values)
			*n* = 46 (9%)	*n* = 62 (12%)	*n* = 213 (40%)	*n* = 209 (39%)	
Influenza vaccines received in 2020–21 season	1.00	Yes	100%	0%	0%	0%	0.000 **
		No	0%	100%	100%	100%	
Actively search for seasonal flu information	0.88	Yes	20%	100%	0%	0%	0.000 **
		No	80%	0%	100%	100%	
Level of involvement in flu vaccination decisions	0.84	Mean score (1 = very low; 7 = very high)	5.41	4.73	4.37	2.40	0.000 **
Attention to seasonal flu information exposed	0.76	Mean score (1 = very low; 7 = very high)	4.61	5.26	4.54	2.70	0.000 **

Chi square/ANOVA tests: * *p* < 0.05, ** *p* < 0.01.

**Table 3:** Sociodemographic characteristics by segment.

Sociodemographic Characteristics		Segment 1 Convinced	Segment 2 Informed Unconvinced	Segment 3 Open to Persuasion	Segment 4 Disengaged Sceptics	Chi Square Test
		*n* = 46 (9%)	*n* = 62 (12%)	*n* = 213 (40%)	*n* = 209 (39%)	(*p*-Values)
Sex	Female	63%	61%	64%	64%	0.986
	Male	37%	39%	36%	36%	
Year of university study	1	46%	48%	47%	41%	0.332
	2	37%	31%	40%	39%	
	3	2%	11%	6%	9%	
	4	15%	10%	7%	11%	
Area of university study	Business	17%	44%	38%	35%	0.047 *
	Science & Engineering	15%	16%	16%	20%	
	Languages	13%	3%	6%	7%	
	Social sciences	35%	19%	17%	22%	
	Tourism & Hospitality	9%	16%	15%	10%	
	Health-related	11%	2%	8%	6%	
Percentile ranks on academic results	C or below (below 80%)	7%	6%	6%	3%	0.395
	B grade (80–90%)	59%	71%	59%	64%	
	A or above (above 90%)	35%	23%	35%	33%	

Chi square tests: * *p* < 0.05, ** *p* < 0.01.

**Table 4:** Personal health conditions and flu vaccine-related family conditions by segment.

Personal Health Conditions and Flu Vaccine-Related Family Conditions			Segment 1 Convinced *n* = 46 (9%)	Segment 2 Informed Unconvinced *n* = 62 (12%)	Segment 3 Open to Persuasion *n* = 213 (40%)	Segment 4 Disengaged Sceptics *n* = 209 (39%)	Chi Square Test (*p*-Values)
Personal health conditions	With chronic diseases	Yes	26%	13%	8%	10%	0.007 **
		No	74%	87%	92%	90%	
	Had received other vaccines voluntarily	Yes	50%	39%	40%	32%	0.073
		No	50%	61%	60%	68%	
	Had severe allergic reaction to vaccine injection	Yes	0%	3%	0%	1%	0.305
		No	100%	97%	100%	99%	
	Frequency of flu-related illness in past 5 years	Every year (5 years)	11%	11%	9%	8%	0.773
		Most years (3–4 years)	11%	5%	10%	8%	
		Once or Twice (1–2 years)	28%	34%	24%	29%	
		Never	50%	50%	57%	55%	
Live with or in frequent contact with …	elderly people	Yes	33%	24%	26%	31%	0.545
		No	67%	76%	74%	69%	
	young children	Yes	17%	19%	18%	12%	0.299
		No	83%	81%	82%	88%	
	People with chronic illness	Yes	15%	13%	17%	25%	0.053
		No	85%	87%	83%	75%	
Family members received flu vaccine in 2020–21 season		All	28%	2%	1%	1%	0.000 **
		At least half	48%	10%	5%	6%	
		Less than half	22%	14%	16%	8%	
		None	2%	74%	78%	85%	

Chi square tests: * *p* < 0.05, ** *p* < 0.01.

**Table 5:** Seasonal influenza vaccine-related behaviours, attitudes, and intentions by segment.

Seasonal Influenza Vaccine-Related Behaviours, Attitudes, and Intentions			Cluster 1 Convinced *n* = 46 (9%)	Cluster 2 Informed Unconvinced *n* = 62 (12%)	Cluster 3 Open to Persuasion *n* = 213 (40%)	Cluster 4 Disengaged Sceptics *n* = 209 (39%)	Chi Square/ANOVA Test (*p*-Values)
Behaviours	Vaccinated in 2020–21 season	Yes	100%	0%	0%	0%	0.000 **
		No	0%	100%	100%	100%	
	Frequency of vaccination in the past 5 years	Every year (5 years)	37%	2%	0%	0%	0.000 **
		Most years (3–4 years)	24%	2%	5%	0%	
		Once or twice (1–2 years)	37%	11%	21%	6%	
		Never	2%	85%	74%	94%	
Attitudes	Confidence	Mean score (1 = very low; 7 = very high)	5.12	3.74	4.16	4.02	0.000 **
	Complacency		2.80	3.54	3.91	4.62	0.000 **
	Convenience		4.57	4.04	3.78	3.73	0.000 **
	Calculation		4.80	4.95	4.93	4.63	0.024 *
	Collective responsibility		4.98	4.65	4.79	4.27	0.000 **
Intentions	Will receive flu vaccine in 2021–22 season	Mean score (1 = definitely would not; 7 = definitely would)	5.27	3.66	3.63	2.17	0.000 **

Chi square/ANOVA tests: * *p* < 0.05, ** *p* < 0.01.

**Table 6:** Seasonal influenza vaccine-related information search, exposure, and trust by segment.

Information Search, Exposure, and Trust			Cluster 1 Convinced *n* = 46 (9%)	Cluster 2 Informed Unconvinced *n* = 62 (12%)	Cluster 3 Open to Persuasion *n* = 213 (40%)	Cluster 4 Disengaged Sceptics *n* = 209 (39%)	Chi Square/ANOVA Test (*p*-Values)
Seasonal influenza-related information search	Actively search for seasonal flu information	Yes	20%	100%	0%	0%	0.000 **
		No	80%	0%	100%	100%	
	Usual channels for flu information search	Search engines	100%	97%			0.458
		Online forums	33%	71%			0.031 *
		Social media sites	56%	86%			0.049 *
		Government websites	67%	48%			0.301
		School clinics	56%	37%			0.295
		Family or friends	78%	87%			0.478
		Teachers	22%	36%			0.418
		Doctors or other healthcare professionals	56%	56%			0.960
	Trust in usual flu information search channels	Mean score (1 = do not trust at all; 7 = completely trust)	5.22	5.23			0.992
Exposure to seasonal influenza-related information or news	Attention to seasonal flu information exposed	Mean score (1 = very low; 7 = very high)	4.61	5.26	4.54	2.70	0.000 **
	Trust in sources from which most flu information was received	Mean score (1 = not trust at all; 7 = completely trust)	5.20	5.12	4.99	4.63	0.000 **

Chi square/ANOVA tests: * *p* < 0.05, ** *p* < 0.01.

**Table 7:** Claimed effectiveness of message in motivating seasonal influenza vaccination by segment.

Promotion Message		Cluster 1 Convinced *n* = 46 (9%)	Cluster 2 Informed Unconvinced *n* = 62 (12%)	Cluster 3 Open to Persuasion *n* = 213 (40%)	Cluster 4 Disengaged Sceptics *n* = 209 (39%)	ANOVA Test (*p*-Values)
Type of message	Promotion focus	4.46	4.36	4.29	3.54	0.000 **
	Prevention focus	4.28	4.32	4.19	3.48	0.000 **
	Incentive focus	4.85	4.30	4.64	3.96	0.000 **
	Individual-oriented	4.26	4.20	4.23	3.55	0.000 **
	Collective-oriented (family & friends)	4.79	4.54	4.51	3.73	0.000 **
	Collective-oriented (community)	4.53	4.40	4.26	3.54	0.000 **

Remarks: 1. Data shows the mean score (1 = definitely would not get vaccinated; 7 = definitely would get vaccinated) 2. ANOVA tests: * *p* < 0.05, ** *p* < 0.01.

**Table 8:** Attitudes, intentions, and behaviours of COVID-19 vaccination by segment.

COVID-19 Vaccination Behaviours, Attitudes, and Intentions			Cluster 1 Convinced *n* = 46 (9%)	Cluster 2 Informed Unconvinced *n* = 62 (12%)	Cluster 3 Open to Persuasion *n* = 213 (40%)	Cluster 4 Disengaged Sceptics *n* = 209 (39%)	Chi Square/ANOVA Test (*p*-Values)
Behaviours	Received COVID-19 vaccine	Yes	30%	13%	6%	8%	0.000 **
		No	70%	87%	94%	92%	
	Family members received COVID-19 vaccine	All	13%	3%	1%	4%	0.000 **
		At least half	17%	10%	14%	9%	
		Less than half	35%	24%	21%	20%	
		None	35%	63%	64%	67%	
Attitudes	Confidence	Mean score (1 = very low; 7 = very high)	3.15	3.19	3.19	2.64	0.000 **
	Complacency		3.12	3.17	3.38	3.30	0.347
	Convenience		4.50	4.85	4.36	4.09	0.002 **
	Calculation		4.71	4.73	4.55	4.27	0.015 *
	Collective responsibility		4.44	4.59	4.33	3.82	0.000 **
	Level of involvement in COVID-19 vaccination decisions	Mean score (1 = very low; 7 = very high)	5.18	5.38	4.92	4.19	0.000 **
Intentions	Will receive COVID-19 vaccine	Mean score (1 = definitely would not; 7 = definitely would)	4.10	3.95	3.98	3.12	0.000 **

Chi square/ANOVA tests: * *p* < 0.05, ** *p* < 0.01.

## Data Availability

The data presented in this study are available on request from the corresponding author.

## Supplementary Materials

The following are available online at https://www.mdpi.com/article/10.3390/vaccines9121397/s1, Supplementary File S1: questionnaire employed in this study.
