# Supplementary material for: Motivating Seasonal Influenza Vaccination and Cross-Promoting COVID-19 Vaccination: An Audience Segmentation Study among University Students
Source: Vaccines (Basel). 2021 Nov 25;9(12):1397. doi: 10.3390/vaccines9121397 (PMC8705827; doi:10.3390/vaccines9121397)
Supplement: Supplementary file 1 [file vaccines-09-01397-s001.zip › vaccines-1482647-supplementary.pdf]

Supplementary Material: Questionnaire

Section (i): sociodemographic and academic details

1. What program are you currently studying?
2. Year of study?
3. What is your current GPA?
4. What is your gender? (Male, Female)
5. What if your age?
6. Do you live with or in frequent contact with … (Yes, No)
    - Elderly people
    - Young children aged below 12
    - People with chronic diseases (e.g. Respiratory conditions such as asthma, diabetes, heart disease) or weakened immune system

Section (ii): personal health conditions, history of influenza-like illness, vaccination behaviours of family members, and experiences associated with other vaccines

7. Do you require medical follow-up and take medication regularly? (Yes, No)
8. Do you have a weakened immune system because of a disease (e.g. cancer) or a medicine that affects your immune system? (Yes, No)
9. Do you have drug allergy? (Yes, No)
10. Have you ever chosen to receive vaccines (e.g. HPV, Hepatitis vaccines) that are not compulsory or required to? (Yes, No)
11. Have you ever encountered severe allergic reaction to vaccine injection? (Yes, No)
12. How frequent do you have flu-related illness the past five years? (Every year (5 years), Most years but not all (3-4 years), Once or Twice (1-2 years), Never)
13. Number of family members (including yourself) received flu vaccination in 2020? (All, At least half to all, Less than half to none, None)

Section (iii): seasonal influenza vaccine-related behaviours, attitudes, and intentions

14. Did you get seasonal flu vaccination in 2020? (Yes, No)
15. What is your frequency of seasonal flu vaccination uptake over the past five years? (Every year (5 years), Most years but not all (3-4 years), Once or Twice (1-2 years), Never)
16. Please rate your **LEVEL of AGREEMENT** with the following statements : (7-point Likert scale: 1-strongly disagree; 7- strongly agree)
    - Seasonal flu vaccine is safe.
    - Seasonal flu vaccination is effective in protecting individuals from getting influenza.
    - Regarding the recommendation of seasonal flu vaccination, I am confident that government and HK healthcare system could decide the best interest of the community.
    - I am concerned about side-effects of seasonal flu vaccines. (reverse scale)
    - Personally, I think seasonal flu vaccination is unnecessary because my immune system is strong enough to fight against the influenza virus
    - Seasonal flu vaccination is not a priority for me because influenza is not a serious illness but just a routine and mild illness
    - If I get a flu, the illness will greatly affect my academic study, social life, or job. (reverse scale)
    - For me, the location to receive seasonal flu vaccinations is convenient.
    - Unavailability of free seasonal flu vaccine is a main barrier to vaccination. (reverse scale).
    - When I think about getting vaccinated, I weigh benefits and risks to make the best decision possible.

- The risks of having seasonal flu vaccinated are lower than the benefits.
- I get flu vaccinated because I can also protect people with a weaker immune system.
- Seasonal flu vaccination is a collective action to prevent the spread of influenza.

17. For me, receiving seasonal flu vaccine is …. (7-point semantic differential scales)
    - Unimportant 1 2 3 4 5 6 7 Important
    - Of no concern 1 2 3 4 5 6 7 Of concern to me
    - Means nothing to me 1 2 3 4 5 6 7 Means a lot to me
    - Does not matter 1 2 3 4 5 6 7 Matters to me
18. What is your intention to be vaccinated in 2021? (7-point Likert scale: 1- definitely would not; 7- definitely would)

Section (iv): seasonal influenza-related information search, exposure, and trust

19. Have you actively searched for information about seasonal flu? (Yes, No) If no, jump to Q5
20. Where do you usually search for information about seasonal flu? (multiple answers)
    - Online search
    - Online forums
    - Social media
    - Government websites
    - School clinics
    - Family members or friends
    - Teachers
    - Doctors or other healthcare professionals
21. Overall, how confident are you that you could get advice or information about seasonal flu if you needed it? (7-point Likert scale: 1- not confident at all; 7 - completely confident).
22. In general, how much attention have you paid to the information or news about seasonal influenza when you are exposed to? (7-point Likert scale: 1- no attention; 7 - a lot of attention)
23. From what source have you received the most information about seasonal flu (multiple answers):
    - TV news
    - Radio
    - Online search
    - Online forums
    - Social media
    - Government websites
    - Emails from school
    - On-campus materials (e.g., poster)
    - School clinics
    - Family members or friends
    - Teachers
    - Doctors or other healthcare professionals
24. Thinking about the source you have received the most seasonal flu information from, [would you say that you] trust information from that source? (7-point Likert scale: 1- do not trust at all; 7 – completely trust).

Section (v): message and influencer in motivating seasonal influenza vaccination

25. How likely will the following promotion message motivate you to receive seasonal flu vaccination? (7-point Likert scale: 1- definitely would not; 7 - definitely would)
- Get flu vaccinated to protect you from flu and its complications
- Get flu vaccinated to protect you and your family from flu and its complications
- Get flu vaccinated to protect you and your friends from flu and its complications
- Get flu vaccinated to protect you and your loved ones from flu and its complications

- Get flu vaccinated to protect you and vulnerable people in the community
- Get flu vaccinated, save lives
- Get flu vaccinated to reduce your risk of flu and its complications
- Get flu vaccinated to reduce risk of flu and its complications for you and your family
- Get flu vaccinated to reduce risk of flu and its complications for you and your friends
- Get flu vaccinated to reduce risk of flu and its complications for you and your loved ones
- Get flu vaccinated to keep flu virus out of our community
- Get flu vaccinated, reduce flu-related complications and deaths in the community
- Stay healthy to achieve the best academic performance during flu season, get vaccinated
- Stay healthy and free of flu-related illness for your dating during flu season, get vaccinated
- Stay healthy during exam period, get vaccinated
- Get a cheaper and faster flu vaccination in campus clinic
- Get a flu vaccination in campus clinic at half price ($180 at school vs >$350 at private clinics)
- Book your flu vaccination by end September to receive a HK$50 early bird discount
- Get flu vaccinated and enjoy a basic body check at discount price.
- Don't let the flu affects your academic performance during flu season, get vaccinated
- Don't let the flu affects your dating during flu season, get vaccinated
- Don't let the flu affects your exam results, get vaccinated

26. How likely will the advice from the following people motivate you to receive seasonal flu vaccination? (7-point Likert scale: 1- definitely would not; 7 - definitely would)
    - Parents & relatives
    - Friends
    - Boyfriend/girlfriend
    - People who share their experiences on forums
    - People who share their experiences on social media
    - Celebrity / KOL
    - Doctors or healthcare professional

<u>Section (vi): COVID-19 vaccination behaviours, attitudes, and intentions</u>

27. Did you receive COVID-19 vaccination? (Yes, No)
28. Number of family members (including yourself) received COVID-19 vaccination? (All, At least half to all, Less than half to none, None)
29. Please rate your **<u>LEVEL of AGREEMENT</u>** with the following statements<u> : (7-point Likert scale: 1-strongly disagree; 7- strongly agree)</u>
    - COVID-19 vaccine is safe.
    - COVID-19 vaccination is effective in protecting individuals from getting coronavirus disease.
    - Regarding the recommendation of COVID-19 vaccination, I am confident that government and HK healthcare system could decide the best interest of the community.
    - I am concerned about side-effects of COVID-19 vaccines. (reverse scale)
    - Personally, I think COVID-19 vaccination is unnecessary because my immune system is strong enough to fight against the coronavirus disease
    - COVID-19 vaccination is not a priority for me because coronavirus disease is not a serious illness but just a routine and mild illness
    - If I get coronavirus disease, the illness will greatly affect my academic study, social life, or job. (reverse scale)
    - For me, the location to receive COVID-19 vaccinations is convenient.

- Unavailability of free COVID-19 vaccine is a main barrier to vaccination. (reverse scale).
- When I think about getting vaccinated, I weigh benefits and risks to make the best decision possible.
- The risks of having COVID-19 vaccinated are lower than the benefits.
- I get COVID-19 vaccinated because I can also protect people with a weaker immune system.
- COVID-19 vaccination is a collective action to prevent the spread of coronavirus disease.

30. For me, receiving COVID-19 vaccine is …. (7-point semantic differential scales)
    - Unimportant 1 2 3 4 5 6 7 Important
    - Of no concern 1 2 3 4 5 6 7 Of concern to me
    - Means nothing to me 1 2 3 4 5 6 7 Means a lot to me
    - Does not matter 1 2 3 4 5 6 7 Matters to me
31. What is your intention to be COVID-19 vaccinated? (7-point Likert scale: 1- definitely would not; 7- definitely would)
32. How likely will you receive COVID-19 vaccination because of the following reasons? (7-point Likert scale: 1- definitely would not; 7- definitely would)
    - Protect yourself from COVID-19
    - Protect yourself and people close to you from COVID-19
    - Required by job (e.g. internship, part-time or full-time job)
    - Want to avoid compulsory quarantine
    - For travelling
    - For lessened dine-out or entertainment restrictions
    - Want to avoid paying for compulsory regular COVID-19 testing
    - Required by school to resume face-to-face classes in campus
    - Show your social responsibility
    - Recommended by family members or relatives
    - Recommended by friends
    - Recommended by healthcare professionals
    - Respond to government's call
    - Lucky draw (e.g. Top 3 winners among ALL HK people who are vaccinated can get HK$1M)
    - Cash prize (e.g. A HK$50 supermarket coupon for anyone who are vaccinated)
